# A reconfiguration of the sex trade: How social and structural changes in eastern Zimbabwe left women involved in sex work and transactional sex more vulnerable

**DOI:** 10.1371/journal.pone.0171916

**Published:** 2017-02-22

**Authors:** Jocelyn Elmes, Morten Skovdal, Kundai Nhongo, Helen Ward, Catherine Campbell, Timothy B. Hallett, Constance Nyamukapa, Peter J. White, Simon Gregson

**Affiliations:** 1 Department of Infectious Disease Epidemiology, School of Public Health, Imperial College London, London, United Kingdom; 2 Department of Public Health, University of Copenhagen, Copenhagen, Denmark; 3 Biomedical Research and Training Institute, Harare, Zimbabwe; 4 Department of Psychological and Behavioural Science, London School of Economics and Political Science, London, United Kingdom; 5 MRC Centre for Outbreak Analysis and Modelling and NIHR Health Protection Research Unit in Modelling Methodology, Department of Infectious Disease Epidemiology, School of Public Health, Imperial College London, London, United Kingdom; 6 Modelling and Economics Unit, National Infection Service, Public Health England, London, United Kingdom; University of Missouri Kansas City, UNITED STATES

## Abstract

Understanding the dynamic nature of sex work is important for explaining the course of HIV epidemics. While health and development interventions targeting sex workers may alter the dynamics of the sex trade in particular localities, little has been done to explore how large-scale social and structural changes, such as economic recessions–outside of the bounds of organizational intervention–may reconfigure social norms and attitudes with regards to sex work. Zimbabwe’s economic collapse in 2009, following a period (2000–2009) of economic decline, within a declining HIV epidemic, provides a unique opportunity to study community perceptions of the impact of socio-economic upheaval on the sex trade. We conducted focus group discussions with 122 community members in rural eastern Zimbabwe in January-February 2009. Groups were homogeneous by gender and occupation and included female sex workers, married women, and men who frequented bars. The focus groups elicited discussion around changes (comparing contemporaneous circumstances in 2009 to their memories of circumstances in 2000) in the demand for, and supply of, paid sex, and how sex workers and clients adapted to these changes, and with what implications for their health and well-being. Transcripts were thematically analyzed. The analysis revealed how changing economic conditions, combined with an increased awareness and fear of HIV–changing norms and local attitudes toward sex work–had altered the demand for commercial sex. In response, sex work dispersed from the bars into the wider community, requiring female sex workers to employ different tactics to attract clients. Hyperinflation meant that sex workers had to accept new forms of payment, including sex-on-credit and commodities. Further impacting the demand for commercial sex work was a poverty-driven increase in transactional sex. The economic upheaval in Zimbabwe effectively reorganized the market for sex by reducing previously dominant forms of commercial sex, while simultaneously providing new opportunities for women to exchange sex in less formal and more risky transactions. Efforts to measure and respond to the contribution of sex work to HIV transmission need to guard against unduly static definitions and consider the changing socioeconomic context and how this can cause shifts in behavior.

## Introduction

Biomedical advances to prevent HIV transmission and a renewed impetus to control the HIV epidemic have led to growing attention of groups within which HIV transmission is still high. Globally, female sex workers (FSWs) have a disproportionate burden of HIV infection[1] and engagement with HIV services is often sub-optimal[2, 3]. HIV risk in sex workers is influenced by various intersecting and interacting social, economic, and political forces that can constrain autonomous and rational decisions over safe sex and impede access to health services[4–7]. Commentators warn that failure to recognize how social and structural factors interface with sex work, could limit the effectiveness of behavioral and biomedical approaches.

Interventions designed to alter the social, political, legal, economic arrangements that determine how power is distributed in society[4], so-called “structural interventions”, are increasingly considered the most appropriate approaches for sustainable HIV prevention among FSWs[7–9]. Such interventions have strengthened evidence of the relationship between the structural determinants and HIV transmission, relationships which are not easily testable in the framework of a randomized control trial[10]. A well-known example, the Sonagachi HIV Intervention Project (SHIP), drove large and sustained increases in condom use by sex workers in Calcutta, India–where HIV prevalence has remained lower than in other states–by targeting key social, economic and cultural structures that disenfranchised sex workers[4, 11, 12]. SHIP facilitated the generation of a sex worker collective that focused on a human and labor rights-based agenda promoting sex work as legitimate employment[4]. The individual and social impacts were sustained by a combined economic and socio-political approach that included micro-credit schemes and reducing stigma and discrimination at multiple levels such as civil society, law enforcement and politicians[4, 7, 9, 11, 13–16].

However, just as countries experience different HIV epidemics, social relations, alliances and political histories also vary[17, 18], therefore it is important to understand how structural forces influence behavior and population health in different contexts[7, 9, 16]. In sex worker populations of sub-Saharan Africa in particular, little is known of the mechanisms of how structural factors alone and synergistically combine to affect health or shape individual behaviors[9, 13, 14]. The collapse of Zimbabwe’s economy in 2009 in the context of a large generalized epidemic with high AIDS mortality[19] provides a rare opportunity to investigate how changes in these social and structural forces may have reconfigured sex work.

### Sex work, HIV, and the economic crisis in Zimbabwe

Between 2000–2008, the economy of Zimbabwe shrank substantially (real GDP declined by over 40%)[20]. A somewhat steady decline in the economy between 1997 and 2005 started to escalate until early 2007, after which point it rapidly capitulated with annual inflation increasing from 2000% to over 500 billion percent in 18 months (March 2007-September 2008)[21, 22]. The replacement of the Zimbabwean dollar with foreign currencies (primarily the US dollar and South African Rand) in February 2009 concluded a decade of economic volatility and record hyperinflation[23]. Declining living standards coincided with large reductions in HIV prevalence from an estimated peak in the general population of 26.5% (uncertainty range: 25.0%-27.8%) in 1997 to 14.7% (13.7%-15.7%) in 2012[24]. A multi-disciplinary analysis of behavioral changes associated with HIV decline between 1997–2007 attributed this, in part, to decreased demand for paid sex among men; findings suggested an increased awareness of HIV risk and limited disposable income as poverty increased both reduced demand[19, 25].

The extremity of the sudden collapse of the economy within a context of a severe HIV epidemic offers a unique set of circumstances to improve understanding about how large-scale social and structural changes–outside of the bounds of organizational intervention–may reconfigure attitudes, behaviors and lived experiences with regards to the wider sex trade. This is the overarching aim of the paper which we address with a large dataset of focus group discussions held in January-February 2009. This cross-sectional study was originally designed within a broader set of objectives to explore the feasibility of conducting a cohort survey of FSWs in rural communities planned for 2010[26]. One of several questions motivating this study was “How had economic collapse, in the context of a declining HIV epidemic, reconfigured social norms, attitudes, lived experiences with regards to the practice of sex work in Manicaland Province?”. As the severity of the economic and social impact emerged so powerfully, we began to emphasize more temporal aspects within later discussions. This provided a rich dataset which was analyzed within a conceptual framework to draw out specific themes relating to the process of social change. The themes emerging from this analysis shed light on a particularly interesting time and place and effectively illustrate how broader structural influences affect the sex trade.

We consider the ‘wider sex trade’ to include transactional sex as well as commercial sex. We note that it can be difficult to distinguish sex work from transactional sex (TS), particularly when social meanings of sexual behavior are changing[27], when acts and motivations are similar[28] and where stigmatization of prostitution inhibits self-identification. In this paper, we identify TS as sex-exchange encounters involving women who do not self-identify as sex workers with no obvious negotiation of payment and/or if payment is seen as a ritual of courtship[28, 29]. While recognizing this distinction between women engaging in TS and sex workers and focusing primarily on commercial sex work, this paper identifies social interconnections between the two.

### Conceptual framework

**Fig 1:**
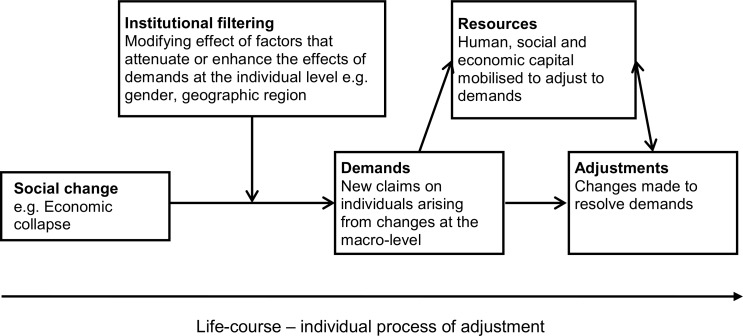
The Jena Model of Social Change and Human Development conceptual framework, adapted for Zimbabwe. The Jena Model of Social Change and Human Development, developed by Silbereisen and Tomasik [30] describes how communities experience and respond actively to the demands of social change. This model was adapted to the Zimbabwe context and is a conceptual framework for how economic collapse might produce changes to sex work organization. The economic crisis placed new demands on individuals (e.g. job loss, food insecurity). Factors such as gender or geographic context can modify the influence of this social change by reducing or enhancing the impact of these demands at the individual level. Resources form part of the process of adaptation to new challenges and mediate individuals’ to meet them. Adjustments are the individual-level and structural changes that occur in response to the macrolevel social change (i.e. economic collapse).

To structure our analysis, we draw on the Jena Model of Social Change and Human Development[30]. According to Silbereisen and Tomasik, uncertainty at the macro-level (e.g. in the economy) “confronts individuals with demands that index a new state of affairs compared to what he or she was accustomed”. These “demands of social change”—i.e. new challenges on individuals that arise through social change (Fig 1)—lie at core of the Jena model.

As individuals attempt to adapt to changing contexts–encountering new demands–they have various resources at their disposal (e.g. human and social capital) which form part of the process of adaptation to the new challenges. The individual-level changes that occurred in response to the socio-economic demands, mediated by individual-level and group-level resources, are the adjustments which ultimately determine the organization of sex work (Fig 1). We used the model to identify individual-level demands, the resources that influence individual attempts at resolving these, and the final adjustments made to resolve them. In addition, community-level constructs (e.g. socio-economic status of the area) can effectively filter the effects of social change thereby modifying the demands felt at the individual level. In this paper, we contrast findings from communities that differ socio-economically to explore whether there was evidence of community-specific structures that may attenuate or enhance adaptive processes at the individual level and consequently influence the nature of sex work.

## Methods

Ethical approval for the study was obtained from the Medical Research Council of Zimbabwe (MRCZ/A/681) and the Imperial College Research Ethics Committee (ICREC_9_3_13).

### Study participants and recruitment

Between January and February 2009, 122 participants across 5 study sites in Manicaland, eastern Zimbabwe, were purposively sampled to participate in 16 focus group discussions (FGD) with the aim of understanding local perspectives on the sex industry (Table 1). Four of the five study sites were chosen because these had been identified as sites for the subsequent cohort study and represented different socio-economic strata: a small town, a roadside trading settlement (RTS), a large-scale forestry estate, and a subsistence farming area (SFA). The growth point (a semi-urban settlement about the size of a small town) was selected as a pilot site based on its proximity to the other four sites. The study communities participate in the wider Manicaland HIV/STD Prevention Cohort Study and project staff had worked previously with local community health workers (CHW) in each area since 1998[31].

**Table 1:** The location and participant composition of the focus group discussions.

Site type	Socio-economic status	Social group^a^	Median age (range)	Number of participants	Recruited by CHW or convenience
*Total participants*			*31 (18–50)*	*122*	
Subsistence farming area	Rural	Males	26 (20–42)	11	Convenience
		Married women	29 (18–33)	8	CHW
		FSWs	34.5 (34–35)	2	CHW
		Overall	27.5 (18–42)	21	-
Roadside trading settlement^b^	Rural	Males	33.5 (25–50)	10	CHW
		Married women	30 (25–45)	8	CHW
		Vendors	N/A^d^	8	Convenience
		Overall	31.5 (25–50)	26	-
Forestry estate	Estate	Males	36 (20–47) ^d^	10	CHW
		Married women	27.5 (19–43)	8	CHW
		FSWs	37.5 (30–49)	8	CHW
		Overall	35 (19–49)	26	-
Small town	Urban	Males	35 (28–40)	6	CHW
		Vendors	35.5 (27–42)	12	Convenience
		FSWs	36.5 (24–50)	10	CHW
		Overall	35.5 (24–50)	28	-
Growth point^c^	Urban	Bar servers	N/A^d^	5	Convenience
		Vendors	24 (18–35)	8	Convenience
		FSWs	27.5 (25–39)^d^	7	CHW
		Married FSW	23	1	CHW
		Overall	26 (18–39)	21	-

^a^Each focus group discussion (FGD) comprised one social group for reasons stated in the text

^b^Missing female sex workers (FSWs)

^c^Pilot area; missing male FGD

^d^Ages of some/all participants were not disclosed

We identified four groups of participants for purposive selection: self-identifying FSWs; men who habitually visited bars or worked in the local commercial center; market vendors/traders; married women (Table 1). We sought diverse female voices to describe the spectrum of sexual exchanges in these communities. Female market vendors and bar workers were chosen because, in many settings globally, they also occasionally sell sex[32–34]. Vendors are also omnipresent in daily community life whilst the perspective of bar assistants provides a useful counterpoint to those of FSWs and males on bar-based sex work. Married women were included to provide a more normative view of sexual relations in order to understand attitudes to commercial (and non-commercial) sex, and of the economic position of women (though we do not analyze this latter topic in this paper). We targeted a wide age range of adults (18–50 years) to capture experiences of those who recently became sexually active, and of individuals with sexual experience throughout the economic crisis (Table 1), but for ethical reasons, all participants had to be 18 years or older.

In each site, we engaged local community health workers (CHW) to assist with recruitment. We relied on CHW knowledge to locate hard-to-access self-identifying FSWs for the FGDs (Table 1; total 4 groups). Where possible (e.g. respondents available in sufficient numbers) and assisted by CHW, we also took convenience samples of groups working in the community centers of each site during the daytime to avoid over-reliance on choices made by CHW (which may bias selection in favor of their social networks)(Table 1).

We did not collect refusal rates from recruitment made through CHW and encountered few refusals when we conducted the convenience samples, though numbers were not recorded. The main reason for refusal was inability to spare time away from work and possibly introduced a selection bias based on employment status or time commitments of respondents. In the RTS, respondents reported that a zero-tolerance campaign on sex workers in bars meant FSWs had become completely hidden; we were unable to find sex worker participants because the health worker did not acknowledge knowing any. Reports on sex work therefore came from market vendors and men, many of whom self-identified as clients of sex workers.

### Focus group discussions

Focus groups were homogeneous by gender, with females further stratified by principal social group based on proximity to the sex trade (i.e. active sellers, omnipresent observers/part-time sellers, non-FSW married women). The groupings were based on anthropological literature about social norms (e.g. tensions exist between female spouses and FSWs) and on advice from experienced researchers from the Biomedical Research and Training Institute (BRTI) in Harare to encourage open discussion[35, 36]. We chose FGDs to allow diverse opinions to be expressed, discussed and contested, providing insight into prevailing social norms and relations[35].

The initial topic guide specifically focused on how sex work is operationalized within a context of extreme economic distress i.e. where FSWs met clients, how regularly they sold sex, how much they charged clients, what relations were like with the wider community and to identify any concerns about recruiting FSWs to a cohort study in small rural communities. However, as the economic decline, and associated community tensions, featured prominently in the initial FGDs piloted in the growth point, we honed in on the participants’ views and perspectives on changes to the demand for, and supply of, paid sex (comparing circumstances in the year 2000 to current circumstances in 2009), including the social and structural changes sex workers and their clients have had to adapt to, and the influence of these changes to their health and well-being. As a result, discussants in the other four sites reflected retrospectively on how sex work differed prior to the crisis compared with the present day. The year 2000 was a suitable reference point, because it was particularly memorable moment when substantial political upheaval coincided with a severe drought[25].

Prompts and questions introduced in the focus groups refrained from asking leading questions about any specific change in sex work, the direction of any possible change, and also the causes of such changes. Due to practical constraints, we were unable to fully explore all emerging themes, however responses to the core topics did reach saturation (i.e. limited new information emerged towards the end) in each site[37].

KN moderated all the discussions in the local language (Shona), which were recorded using a digital Dictaphone. JE was present in all FGDs but played no active role; discussions were not translated in situ to avoid disrupting the discussions. As an experienced researcher with BRTI, KN was able to engage with the participants and make them feel at ease while allowing them time to share and discuss, but also probe for differing or dissenting views. Respondents were members of communities that were well-acquainted with BRTI and the cohort study, therefore any association with HIV prevention and condom promotion was unavoidable; we expected this could bias responses to be more socially desirable.

FGDs took place in neutral, discreet locations within the study sites, including hotels and CHW houses. In 2/5 sites a male nurse was present to answer medical questions. The goals of the FGD were explained to participants in the first ten minutes, and individuals who wanted to participate provided signed informed consent (no-one left at this stage). We explained that we were hoping to understand about men and women’s social activities and why some women may get involved in sex work. This included wanting to understand where these women work, particular practices, their integration into the community and community attitudes about sex work. Active participation was encouraged, but responses were voluntary. Participants were asked to use pseudonyms if they wanted to explain events or situations that had occurred to themselves or friends. In recognition for their time, each focus group member was given one bar of laundry soap; two bars were given if the discussion exceeded one hour. Gift amounts were guided by those given by another Manicaland Project team conducting research contemporaneously, using similar methods and in similar communities[38].

### Data analysis

In total, 16 transcripts were transcribed and translated verbatim by KN. Transcripts were written in Microsoft Word. Data were analyzed inductively by JE using thematic network analysis[39] and Nvivo8 software (QSR International). Thematic network analysis is a flexible analysis of text data occurring over several stages of coding[39]. We used inductive coding “to provide a rich thematic description” of the data, identifying dominant and salient themes[40]. An initial code list was developed from the longest transcripts in each site to represent distinct ideas describing how sex work was identified, operationalized and impacted by socio-economic forces. This formed the preliminary framework which was then checked for duplicates and validated, by applying it to the other transcripts, and refined to create 34 unique codes.

The Jena Model was then used to further focus our coding framework by re-reading all the transcripts to identify which codes met each component of the model process: the demand, the resources, and the adjustments/adaptations (Fig 1). Once the codes were organized, text segments for each code were extracted. Segments were re-read as themes and then grouped on the basis of similar concepts and organizing ideas (Table 2). The transcripts were then re-read to ensure that these concepts and organizing ideas were consistent with the data. We identified structural changes which emerged from the specific demands that impacted sex work and which were created by the macro-level socio-economic changes.

**Table 2:** Thematic analysis of how social and structural changes in Zimbabwe have had an impact sex work.

Codes	Issues discussed	Basic themes	Organizing themes
- Fluctuating affordability	- High demand from “Chiadzwa diamond” panners Economic decline: No money for drinking or buying sex	1. Local and national economic fluctuations alter demand for paid sex	The changing context of the sex trade in eastern Zimbabwe
- Income earned in baskets of goods	- Income on the estate was paid in commodities		
- Piece-meal jobs	- Irregular pay days created irregular demand		
- Drinking rituals	- “Binge-drinking” before sex negotiation Opportunistic purchase of sex		
- Fear	- Sex work associated with HIV Awareness of disease stopped men paying for sex	2. Local social forces reduce demand for paid sex	
- Zero-tolerance policy	- Local policy introduced to tackle HIV: remove women from bars		
- Bar closures Dispersal away from bars	- bar-FSW can be picked anywhere (shopping centers; sex work is in the workplace; women meet clients at funerals/churches) vendors are also FSW; Bar FSWs adjust timing and frequency to compete with vendors	3. Sex negotiation dispersed from visible locations into the community	FSWs and clients adapt to social and structural changes, reconfiguring the sex trade
- Tactics Ways FSWs attract clients	- Knowing your competition Use of body language	4. FSWs employ new interpersonal strategies to recruit clients	
- Deflation/ inflation of prices	- Payments decreased because receive what men can afford; high payments (USD100) during Chiadzwa	5. New forms of payment	
- Change to payment structures	- Payments: sex-on-credit; payment in commodities		
- Poverty fuels TS (e.g. shortage of remittances)	- (Financial; lack; emotional; retaliation; survival/poverty; children; hunger; peer pressure)	6. Social and structural changes have led to an increase in transactional sex	Increased extent and forms of transactional sex
- Wealth inequalities	- Inequalities mean impoverished women need to do TS		
- symbolism of condoms	- Associated with promiscuity	7. Increased transactional sex perceived to fuel the HIV epidemic	
- condoms are not used in TS	- Married women involved in TS are not sex workers		

Due to the difficulty of accessing FSWs, we included information from the pilot focus groups in the growth point to strengthen the interpretation of experiences of FSWs. To preserve the anonymity of the respondents, names of individuals were changed and place names were removed. Quotes were edited for grammar and clarity, while preserving the meaning[41]; an ellipsis indicates text has been removed. Text in square brackets within quotes was added to clarify or to anonymize the material. Unedited, but anonymized, passages are available in the supplementary information (S1 Text).

## Results

In the results that follow, we examine three interrelated organizing themes emerging from our analysis (see Table 2), which, when combined, illustrate how social and structural changes–directly and indirectly–have reconfigured the sex industry in eastern Zimbabwe. Guided by the structure in Table 2, we begin by illustrating how social and structural changes in Zimbabwe had changed the context for sex work (new demands, Fig 1). We then present data illustrating how FSWs and their clients adapted to this new context, reconfiguring the praxis of sex work (resources and adjustments, Fig 1). We end the section by presenting data which suggests that social and structural changes had not only encouraged FSWs and their clients to adapt to a new context, but have contributed to multiple, and at times, extortionate, forms of transactional sex.

### The changing context of the sex trade in eastern Zimbabwe

#### Local and national economic fluctuations alter demand for paid sex

Discussions in all areas were heavily influenced by the effects of a volatile local economy. It was evident in all of our FGDs that economic conditions directly affected demand for paid sex by impacting the affordability of commercial sex and indirectly by affecting the affordability of drinking patterns associated with buying sex. Described experiences predominantly featured the felt effects of a deepening economic crisis that culminated in collapse of the currency in 2009, coinciding with these FGDs. However, in the RTS, the small town and the growth point, economic conditions were also altered by a localized economic boom caused by unregulated diamond panning, colloquially termed the “Chiadzwa period”, which immediately preceded the economic collapse. News reports at the time suggested the mine was left unoccupied between 2006 and 2008, and attracted many unauthorized diamond panners[42] until government authorities claimed control in late 2008[43].

Most respondents in the affected areas mentioned how the commercial sex industry was “resuscitated” by diamond panners who temporarily distorted local monetary systems (it was unclear whether these were local men or non-locals passing through—in the growth point they appeared to be the latter, S2 Text). Daniel, a man from RTS, notes the fluctuating demand in a discussion about the demand for sex work:

Daniel: “I started working in the late 80s and early 90s. I would say that we had our piece of cake, we really enjoyed ourselves … People used to do it [sex work] long back when the economy was still okay. Sex work is going down, it was just resuscitated for a short time during the Chiadzwa diamond days” (Male, RTS)

The diamond panners not only resuscitated the demand, they also stimulated a hike in prices. In a discussion about the earning potential of sex work, a group of FSWs reminisce on the days when prices for sex work were twenty-fold higher than what they received after panners had left.

Hannah: “The time of the diamond panners, we really made money, because they would pay up to about US$100–150 per session … The diamonds panners had a lot of money; they would even give you such an amount for free … without asking for sex.”

KN: “How much are you making these days since the diamond panners are no longer here?”

Charity (and Esther agreeing): “Aah these days it’s difficult, people no longer have money, it’s by chance [that you will find a wealthy client].” (FSWs, growth point)

As Charity and Esther acknowledge, the post-boom austerity was a stark contrast for women who had profited from sex work; not only did prices change, a number of FSWs and clients reported changes to the timing and frequency of demand. Where previously demand fluctuated in a predictable manner–peaking at weekends and around payday when men would typically go to bars–economic decline reduced opportunities for regular employment, which meant that local men could no longer afford drinking in bars or to pay for sex. FSWs and men talked about how the demand for paid sex had become erratic and men would purchase sex on an ad hoc basis if, for example, they had earned money or commodities through piece-work.

Hugh: “Nowadays there is no longer month-end [payday] as far as things are concerned, it depends on what you earn on that day. It can be any income that you managed to receive on that day, whether it is groceries, a bar of soap, maize-meal or whatever you earn that particular day. These FSWs accept anything you have as payment.” (Male, small town)

Noted by Chris below, reduced disposable income also appeared to indirectly vitiate demand for paid sex by interrupting ritualized socializing–characterized by heavy drinking in a male-dominated environment–that was once central to the way sex was negotiated. Male respondents described how such socializing in combination with the attention given to them by FSWs, heightened their sexual arousal which they described as often culminating in unplanned or even involuntarily purchase of sex (S3 Text).

Chris: “For someone to engage with sex workers it means you have a lot of money; people would invite each other and go for a beer binge-drinking. If he has money on a particular day he will call a lot of friends to go and drink beer. That’s what these young men do. But, nowadays, money is scarce. It’s rare for someone to get money and go and drink, because life now is very tough. He would rather stay at home.” (Male, SFA)

Bars thus formed a nexus for sex work by combining the social and chemical properties of drinking, with the socio-cultural reasons that make bars male-dominated (also documented in other studies[44, 45]). Reduced drinking and patronage of bars, due to erratic income, removed an important space for sex negotiations.

#### Social changes alter demand for paid sex

Besides a volatile local economy, several social changes were also observed to impact demand for commercial sex. Fear of HIV and death had become linked to commercial sex and promiscuity more widely.

Rose: “Prostitutes make our lives difficult here. They infect our boyfriends before we start having our own families, so they spoil everything for us. These days young men are eager to have an experience with these old ladies, these prostitutes, so it will be difficult to marry someone who is not infected.” (Vendor, Growth point)

In the RTS community, focus group participants indicated an escalation of intolerance against selling sex in bars. The following exchange highlights how anxiety about HIV and recognition of the need to reduce high-risk behaviors, governed the demand for sex work through public humiliation for purchasing sex and scapegoating of sex workers:

Walter: “Local women used to enter the bars, but we ended up chasing away women from the bars; we didn’t want them in the bars anymore”

KN: “Was it unanimously decided that as a community you agreed not to allow women in the bars?”

Walter: “To answer your question, I would say that many people died. People had a nasty experience due to this disease, so they do not want it to befall them. At funerals, people were aware of the deceased’s previous activities and if s/he was not straight [i.e. if they were promiscuous], then people would warn those remaining to be watchful, for their time will come. People were afraid and they exercised self-control.”

Peter: “Plus these women used to document all the partners they had slept with. They left a record listing all people who had had sex with her.”

KN: “Who would know of what was in the record?”

Michael: “The whole village, the list would be read in public”

KN: “What if your wife was there? Wouldn’t she have heard about that?”

Michael: “Of course, she would know of all your hidden activities!” (Males, RTS)

This scapegoating of FSWs as responsible for HIV transmission seemed to be recognized by FSWs as a perception that they were less “healthy” than women involved in TS. That this perception prevailed, despite their reported insistence on condom use, was a source of frustration for Grace and other FSWs in the small town and growth point who also argued that some men even requested protection (some FSWs met resistance on occasion or men offering more money for unprotected sex, S4 Text). Men in the town and RTS also corroborated this “rule” of condom use for commercial sex, unless they were drunk (S4 Text).

Grace: “Pure prostitutes who are doing it on full time, they use condoms, no matter what. Even if they know that they are HIV/AIDS positive, they know the rules and they use condoms strictly. But for those who are doing it outside [the bars including women involved in TS–see S1 Text for full quote], they are tricky, they do not use condoms, men believe that they are healthy…such people are the ones who spread HIV/AIDS on a fast rate, because they are still trusted. As for us, we are no longer trusted and we now have a number plate, no one trusts us anymore.” (FSW, small town)

It is evident that both the economic decline and fear of HIV has had direct and indirect impacts on the demand for paid sex. We will discuss in the next section how FSWs and their clients have adapted to these changes. In the final section, we return to discuss tensions between TS and sex workers highlighted by Grace and consider reasons for and community reactions to the perceived growth in TS.

### FSWs and clients adapt to social and structural changes, reconfiguring the sex trade

How did FSWs and clients adapt to the new social and economic realities described above, reconfiguring the sex trade? Three strategic changes were identified: change of location, change in interpersonal strategies for attracting clients, and change in monetary systems underlying the transaction.

#### Sex negotiation dispersed from visible locations into the community

In all sites, FSWs and clients mentioned the waning dominance of bars as a locus for commercial sex, but the extent of dispersal away from bars varied by site. In the growth point and small town sites, FSWs continued visiting bars, but with decreased frequency and would look for other opportunities to make money. For example, FSWs who had made money during the Chiadzwa period were “beginning to sell those things they got from that time” (S5 Text). In the other three sites, there was consensus that bar-based solicitation had virtually ceased (as Paul suggests below).

Paul: “In the past, women used to fill the bars and men will fight over women in the bars … Nowadays they are no longer doing that, you find them doing sex work from their homes and from the work place, not in the bars as before. If you go to the bars you would think that there are no sex workers here yet there are there, but they are doing it in their homes and in the bushes, not in the bars anymore.” (Male, estate)

New and alternative places for client recruitment included workplaces, on the roads, in the bushes, and in haulage trucks of drivers delivering to the forestry estate. In the forestry estate, participants reported it was common for negotiations to occur during working hours with sex exchanged after work (see S6 Text). Also, events or gatherings where men and women congregate and mix were mentioned by most focus groups, to provide new avenues for negotiating sex work. This included Chenura ceremonies (celebrations of ancestors that take place in August), during grain distributions from international aid organizations, all-night Church meetings, concerts, weddings and funerals. Christina and Sylvia, two sex workers who used to travel to a nearby growth point to sell sex, explained how the economic decline meant they had to find other opportunities to attract clients.

Christina: “Nowadays it is rare that we meet the clients at the beer halls. We meet them at our homes, as people no longer go to the beer halls.”

Sylvia: “We also see each other at traditional ceremonies to mark the death of a departed family member (Chenura) …”

Christina: “… and at the consolation ceremonies (Nyaradzo).”

Sylvia: “From the way I dance, they [men] will definitely notice me.” (FSWs, SFA)

Third parties and word-of-mouth, as Mary describes above, were also mentioned as ways to find clients in the RTS, though the zero-tolerance policy combined with economic decline appeared to have driven sex work underground. To facilitate clandestine negotiations, FSWs were said to live in accommodation nearby the bar and find non-local clients among the migrant NGO workers whose central distribution point and workers’ accommodation were slightly secluded from, but near the marketplace (we confirmed this with the workers when we visited the distribution point; they referred to local women who brewed “Ndari”–a “seven-day” beer–who also exchanged sex). Jeffrey and market vendors, Mavis and Victoria, shared their observations of the sex work dynamics in the area.

Mavis: “Nowadays, the sex workers are concentrated at that organization that is distributing grain … The sex workers are targeting truck drivers that are bringing in trucks of food there.”

Victoria: “The shop assistants know very well the dynamics that are going on there. They are the ones that are sent to call sex workers by these truck drivers.” (Vendors, RTS)

Jeffrey: “Some of these sex workers know people’s pay days, they know the companies and the pay days, so they usually grab the opportunity [to sell sex then] … We have some non-governmental organizations operating in here, so they keep a record of their pay days” (Males, RTS)

#### FSWs employ new interpersonal strategies to recruit clients

FSWs working in bars in competition with other women developed a range of strategies to attract men and to maximize business, including exploiting fear of HIV and manipulating local perceptions of risk:

Esther: “Older women are very jealous of the younger girls; they will tell the men in the bar that the younger women are infected by HIV/AIDS, so men shouldn’t pick them” (FSW, growth point)

Tactics to help initiate conversations in bars, such as approaching men with an empty cup (to request beer), were less subtle than the sophisticated signals FSWs employed to recruit clients outside bars, as the environment differed substantially from one where solicitation was the norm. FSWs from all groups mentioned having to use a host of body language techniques and linguistic skills to signal that they were approachable (it is culturally unacceptable for women to proposition men with sex directly). Signals ranged from verbal cues to more subtle eye contact and body movements.

Chloe: “Before I became a sex worker, I used to be bothered about how I would indicate that I am approachable. Have you seen those very large hooped earrings? They are called “do not hesitate to approach me!” When you are wearing those earrings, men will know that you are approachable. Also from the way I talk to a man, for instance at a bus terminus, he should know that I am free. I would go and approach him and ask him seductively, ‘Was there any bus that has passed so far?’ (standing up to demonstrate, twisting her body). My posture would also indicate that I am asking for more. If I am a decent person, I would be talking to him more politely, ‘My son-in law, was there any transport here? I am coming from a funeral’. As a sex worker, my manner is completely different. The guy I’m talking to would definitely know that I am free (All laughing from the gestures). It’s called marketing yourself!” (FSW, estate)

#### Changes to monetary systems

All focus group discussions alluded to the elasticity in payments for sex. Hyperinflation and shortage of funds among clients meant changes in monetary systems. While FSWs in the small town and growth point and male focus groups in the RTS and small town talked about how FSWs were accepting smaller payments, some discussed how it was now possible to pay for sex using commodities.

Peter: “Nowadays the market is low, payments are low, ever since the mine was closed.”

Unidentified participant: “If they get groceries then they are paid. They get a piece of bath soap. They can be given a bottle of cooking oil or anything as a form of payment.” (Males, RTS)

This was corroborated by FSWs who reported agreeing to credit-based arrangements, where payment is negotiated upfront, but honored after an agreed period. This emerged as a particularly common form of payment in the forestry estate. A number of features of the estate appeared to contribute to its viability: a) wages were paid in commodities by the proprietary company and thus may have been less amenable to per-encounter payments; b) sex workers and men worked in the same place so FSWs knew how much and when their clients would be paid (and where they lived); c) the relatively stable male workforce meant FSWs knew where to find them to collect payments (few men occupied the low-skilled jobs that involved seasonal rotation and had high turnover; these were some of the only jobs available to women). However, such arrangements still exposed FSWs to extortion by clients who could refuse to pay, as described by FSWs from the forestry estate below.

Phoebe: “Here we get our diamond [hampers given as part of the salary] at the end of the month, that’s when we pay each other…If the company takes two months without paying us, we will then be having sex with each other for those two months for nothing. So, that’s the deal…usually it’s 2 liters of cooking oil and a bar of soap…”

Chloe: “…or 4 liters and a bar of soap”

Phoebe: “If he runs away without paying I would definitely not let him come back again for such services… But I would not make a follow up, it would just mean the end of the relationship… If I am tricked, I am tricked; it is not possible to make a follow up, is it?”

Chloe: “I would try to make a follow up, aah!”

Claris: “He would have used me for nothing, so I would make a follow up until he pays me my money or whatever we have agreed. As soon as he pays me, I won’t bother him anymore or even to bother his new partner, I won’t. I would leave him and look for someone else.” (FSWs, estate)

### Increased extent and forms of transactional sex

The changing social and economic contexts not only had an impact on the practices of existing FSWs to maintain their income through the aforementioned adaptations, but it was also perceived to have affected the wider sex trade and contemporary influx of women and children engaging in transactional sex (TS). Local opinions on the perceived rise in TS also concerned the implications for HIV spread.

#### Transactional sex fueled by poverty

The prosperity of the ‘Chiadzwa period’ against a backdrop of general economic decline was perceived to provide a powerful incentive to sell sex and was noted for an initial spurt in TS including among married women (despite the risk of marital breakdown):

Tiffany: “When we meet as women and we rebuke each other about this behavior, one will say, ‘The man you are chiding me about brings food at home, my own husband doesn’t bring food for the children’ … The husband is just sat at home doing nothing, so the wife will go [to earn], for example, recently, some went to Chiadzwa…She would have gone with the diamond panniers and after a few days she brings home a lot of goods. You observe that everything worked out well, even the family looks well” (Married woman involved in TS, growth point)

However, it was the austerity of the post-boom period, marked by deep poverty and the lack of basic household supplies, that was mentioned in many of the FGDs (including FGDs in communities that had encountered the diamond boom) as the principal contemporary driving factor for housewives to engage in TS, as Moses describes below.

Moses: “Right now because we have a lot of poverty, we have poverty driven sex work [transactional sex]. For [women] who are poverty driven [to sell sex] they maybe in a married couple and the man cannot support their families. The wife will then approach you asking for grain/maize or anything to feed their families and in return they will agree to have sex with you.” (Male, small town)

Moses’ excerpt also illustrates the uneven effects of the economic decline causing women from impoverished households to resort to TS to support the family and who approach men in households that are less impoverished. Because of the cash crisis, and a desire for consumables, several respondents (such as John below and S7 Text) pointed to a rise in transactional sex among adolescents, particularly those from poorer households or orphans.

KN: What you can say about the trend of sex work, how has sex work changed since 2000 and before as you compare it with nowadays?

John: “I wanted to refer to Chris who said sex work is decreasing; I disagree, I believe sex work is increasing. I spend most of my time at the shopping center in my barber shop. I will shave the hair of six girls and, after finishing, they will tell me that they do not have any money to pay for the service … I will see Revai at the shopping center with one girlfriend, he has two or three now, I will see Ruvimbo with the same girlfriend, I will see a third person with the same girlfriend, so I can say sex work is on the increase.” (Male, SFA)

Beatrice: “These children nowadays depend from what they get from men from the bars. That’s where they get sugar, soap, and all other basics.” (FSW, estate)

The combination of sex workers selling sex on a more ad hoc basis and outside bars (leading to a perception of commercial sex diminishing) with a rise in women and children engaging in TS suggests a shifting sex trade.

#### Increased transactional sex perceived to fuel the HIV epidemic

We have described how FSWs were perceived to be responsible for HIV spread despite a tendency for condom use in commercial sex. This trend helped illuminate a perceived difference between commercial sex workers and women who engaged in TS. In discussions about differences between commercial and transactional sex, it often emerged that condoms were less often used in TS partly because men perceived women or girls involved in TS as uninfected (mentioned in every FGD with men). On the other hand, many women considered commercial sex workers less risky, precisely because, unlike women involved in TS, FSWs were believed to use condoms consistently:

Brian: “Some people still have a wrong perception about condoms. There are still some people who believe that condoms are for commercial sex workers. So as a result, if someone falls for somebody’s wife they think that she doesn’t have any infection, they do not use a condom and they also believe that a condom is only used for a sex worker. Again, a married woman would prefer not to use a condom for she doesn’t consider what she is doing as sex work” (Male, forestry estate)

KN: “So what are your views on sex work and sex workers?”

Violet: “I am more pained by married women who are doing sex work than by those who are doing full time sex work in the bars. These married women are spreading HIV/AIDS like a veld fire [wildfire] and it’s a concern to us, but those who are in the bars doing commercial sex work use protection. I do not know what can be done about the married women, for these are the trouble causers.” (Vendor, small town)

We note that Violet’s views contrast with felt experiences of FSWs, that they were “no longer trusted”, and community responses to FSWs (theme 1) and thus possibly reflect more nascent views rather than an established norm (similar views were found in married women’s FGDs in the estate and RTS; though there were dissenting views in the RTS). While all FGDs acknowledged condoms were rarely used in TS encounters, suggestions by males that women may be affronted by requests to use condoms were not corroborated in discussions with females. Rather, they felt the main barriers to condom use concerned the male partner who determined condom use (young girls were widely reported as particularly vulnerable), the risk of losing payment, the unplanned/opportunistic nature of sex, and the fear of discovery by their spouses. If condoms were used, it was only in the first few encounters.

Violet and Brian’s views indicate how local social attitudes to sex work may be shifted by changes in the market for sex. Women–whether involved in sex work or transactional sex–not men were, however, repeatedly held responsible for HIV transmission.

## Discussion

**Fig 2:**
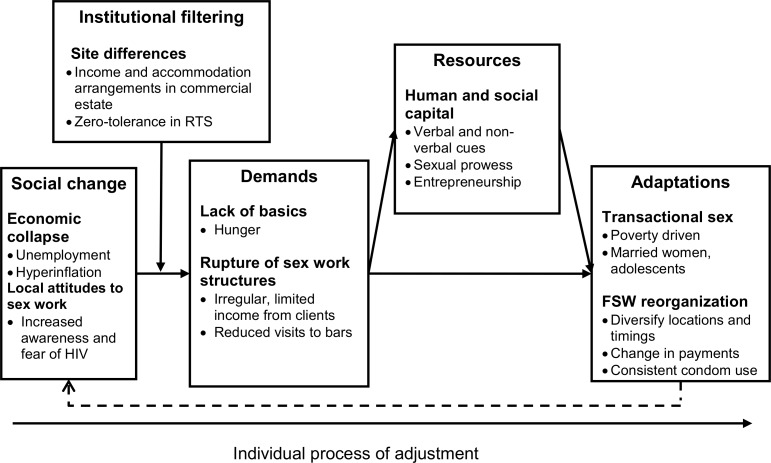
A theoretical framework for the change in sex work in eastern Zimbabwe during the economic crisis. Solid arrows indicate a pathway of adjustment due to social and economic changes; the dashed arrow reflects the shift of local attitudes to the increase in transactional sex (TS). Social change: Economic collapse caused widespread unemployment from both formal and informal sectors. Hyperinflation meant that money ceased to have any value and a barter economy developed. Widespread fear of HIV resulted in scapegoating FSWs. Demands: Men in all communities suffered from the drop in regular income; they reduced visits to bars and visits to female sex workers (FSWs), creating a shortage of client demand for paid sex. Poverty, particularly in poorer households, intensified causing hunger. Institutional filtering: On the commercial forestry estate sex work was sustained because of continued pay, the campus-style onsite accommodation, migrant labor, inequality in employment opportunities and income between the sexes. In the roadside trading settlement (RTS), the community had taken a zero-tolerance approach to FSWs, banning them from bars. As a consequence, sex work was no longer conducted openly. Resources: FSWs met clients outside of bars and developed strategies to indicate they were available for sex. Adjustments: Growth in transactional sex among non-FSWs due to poverty. Prevailing social and economic conditions caused adjustments to the timing, location, terms (e.g. condom use and payments) of transactions. Dashed arrow: Rise in TS (where condom use was not as tolerated because of its association with sex work) shifted local attitudes towards FSWs who were seen as more responsible for using condoms.

The analysis set out to examine how HIV and the economic crisis in Zimbabwe has contributed to a reconfiguration of social norms, attitudes and lived experiences with regards to the practice of sex work in Manicaland Province. Our findings highlight three interrelated structural influences occurring over a period of time: changes in economic conditions; changes in the social norms and attitudes toward sex work; changes in monetary systems. Together these influences illustrate a process of social adjustment, involving major shifts in the sex trade (see Fig 2). Our study exemplifies how a changing socio-economic environment may directly and indirectly influence sex work and contributes to the dearth of data on structural determinants of risk behaviors in FSWs in sub-Saharan Africa[8].

More specifically, and as captured in Fig 2, we found that local and national social and economic changes were perceived to influence the demand for commercial sex. The temporary surge in sex work during the diamond boom was countered by secular economic decline and a wider fear of HIV. These two attenuating factors combined to produce erratic client demands for paid sex and reduced opportunities for FSWs to sell sex in bars. Research conducted in Zimbabwe in the 1990s illustrated that regular demand and known loci for finding sex workers were two integral components of commercial sex. In this period, FSWs mostly worked from drinking venues[46, 47], with business relatively predictably growing towards the end of the week and peaking with “month-end” paydays[46]. Our findings suggest however, that confronted with new socio-economic realities in the late 2000s, FSWs adapted by diversifying sex negotiation spaces (changes to the locations and timing of sex negotiation) and employed different strategies (i.e. manipulating local perceptions of risk and seduction tactics) to recruit clients. The hyperinflationary environment and shortage of cash effectively disenfranchised overt FSWs, forcing them to accept in-kind payments and credit-based arrangements. Finally, an increase in transactional sex altered the sex trade more generally and shifted local attitudes on commercial sex workers, making them stand out as responsible condom users.

In seeking to explain the HIV decline between 1997–2007, Muchini et al, found that awareness of HIV and, secondarily, economic forces drove changes in risk behavior[25]. However, the extremity of the economic collapse that coincided with our study differentiates it from the earlier, more steady secular changes underlying Muchini et al’s findings. Our study was therefore ideally situated as a case study to identify how economic forces in particular may influence the sex trade.

By also addressing the wider market for sex that encompasses both transactional sex (TS) and sex work, we observed seemingly contradictory trends that appeared to fuel tensions between FSWs and women involved in TS. For example, while diamond panning was a stimulus for both sex work and TS, unlike commercial sex, TS appeared not to decrease during further economic decline. Rather, the non-uniform impacts of economic collapse created new opportunities for impoverished women and girls to sell sex for survival and is consistent with quantitative data from the same communities that indicated the poorest communities were worst affected[48]. Secondly, men had become increasingly fearful of sex with FSWs due to high HIV infection rates yet held widespread misconceptions that women/girls involved in TS were uninfected and condom use appeared to be more accepted in commercial sex, but not for individuals involved in TS. Taken together, these findings illustrate how simultaneous poverty-fueled increases in largely unprotected TS may thus have absorbed some of the previous demand for commercial sex, as some sex workers in the small town reported, and raises concerns about the welfare of young women involved in TS and risk for HIV acquisition[49]. Research into sex work or transactional sex has so far predominantly sought to distinguish TS as a form of material exchange distinct from sex work[28, 50], tending to overlook the existence of TS within a wider market for sex and what implications this has for HIV prevention. The dynamics of socio-economic forces, such as the feedback of social processes (e.g. the association of condom use with sex work and subsequent reluctance of men to use condoms in TS), suggests that further work is needed to identify the tensions and dynamics of their co-occurrence[51]. Additionally, widespread scapegoating of women involved in the wider trade of sex (while absolving clients of responsibility) cautions against isolated individual-level HIV interventions that fail to address long-lived taboos around female promiscuity and gendered attitudes[52–54].

Combined with declining demand, the hyperinflationary environment caused changes in the payment systems for sex work and changes in working conditions. Price deflation, commodity payments, providing sex on credit, relying on word-of-mouth or repeat clients increased the financial vulnerability of sex workers and exposed some to extortion. However, we found no definitive evidence for increased risky commercial sex as a result of financial insecurity that has been reported elsewhere[55]. To our knowledge this is the first study to report in any depth on commercialized sex-on-credit arrangements in sub-Saharan Africa, which previously has only been briefly noted[56, 57]. Human capital appeared pivotal in negotiating and ensuring payment from credit arrangements, supporting quantitative research that found a positive association of education level and negotiated price[26].

In Zimbabwe, bars have frequently been used for condom promotion and peer education interventions with sex workers[58, 59] and been advocated for interventions by more recent studies[60]. However, in these predominantly rural communities where spaces for sex negotiation are highly variable, our findings concur with experiences elsewhere[61] and suggest solely venue-dependent approaches to prevention are likely to be of limited effectiveness and sustainability. Our research also suggests the locations where surveys are conducted may affect the apparent size and characteristics of the FSW population and that the fluidity of sex worker transactions can make categorical distinctions for sex work used in surveys (e.g. receiving cash per encounter) uninformative if not adapted to the local context. Therefore, locally relevant definitions of sex work should be considered when modelling and evaluating the contribution of sex work to HIV epidemics[62]. Although our findings are consistent with the decline in HIV incidence associated with declines in casual sex in Zimbabwe and Tanzania[63, 64], this paper stresses the importance of identifying dynamic changes in the wider trade in sex and ensuring their inclusion in HIV modelling and analysis of risk. Finally, interventions need to take into account the social norms of discrimination against women when their livelihoods “challenge ideological constructions of womanhood”[65] and to address possible perverse social forces in the wider trade in sex to improve the lives of both sex workers and women involved in TS.

Our study needs to be evaluated with a number of limitations in mind. Our research team is well-known in study sites for HIV prevention and condom promotion; hence, facilitator effects could have biased responses in favor of socially desirable answers. By developing questions iteratively, we could test the credibility of various statements (e.g. reports on condom use by FSWs were corroborated by males across all sites). One of the main issues for this study was the reliance on participants’ recalling accurately the situation up to ten years previously in relation to their current circumstances. For example, the timing of the study may have caused participants to attribute changes in sex work organization to the economic crisis rather than to social factors. To help recall we used a salient reference point (the year 2000) used elsewhere[25] and we observed considerable internal consistency across FGDs on the overall causes (including social factors), timing and impacts (e.g. amounts paid for sex). The breadth of experiences of FSWs may be compromised by the lack of FSW participants younger than 24 years and our inability to find FSW voices in the RTS (limiting our understanding of the lived experiences of FSWs under the zero-tolerance policy). However, by capturing opinions from clients, FSWs and the wider community we identified a changing social context and its impact on those involved in the sex trade. Furthermore, conducted at the nadir of the economic crisis in early 2009, this study was ideally timed to capture contemporary lived experiences of financial hardship on the local sex industry.

## Conclusion

This paper presented a case study of events in a specific place and over a period of time that showed how large-scale socio-economic changes characterized by three structural influences–changes in economic conditions; changes in the social norms and attitudes toward sex work; changes in monetary systems–reorganized the market for sex and the practice of sex work. These changes reduced previously dominant forms of commercial sex, while simultaneously affording new opportunities for women and adolescents to exchange sex in less formal transactions. These divergent trends are both likely to affect risk of HIV transmission and acquisition. The implications of these dynamics for HIV epidemics need to be better understood.

## Supporting information

S1 Text Unedited quotes. All quotes that are used in the main text are supplied in full and without edits (the texts remain anonymized).
(DOC)

S2 Text Supplementary quotes. (DOCX)

S3 Text Supplementary quote. (DOCX)

S4 Text Supplementary quotes. (DOCX)

S5 Text Supplementary quotes. (DOCX)

S6 Text Supplementary quotes. (DOCX)

S7 Text Supplementary quotes. (DOCX)

## References

[1] Baral S, Beyrer C, Muessig K, Poteat T, Wirtz AL, Decker MR, et al Burden of HIV among female sex workers in low-income and middle-income countries: a systematic review and meta-analysis. Lancet Infect Dis. 2012;12:538–49. 10.1016/S1473-3099(12)70066-X 22424777

[2] Cowan FM, Mtetwa S, Davey C, Fearon E, Dirawo J, Wong-Gruenwald R, et al Engagement with HIV Prevention Treatment and Care among Female Sex Workers in Zimbabwe: a Respondent Driven Sampling Survey. PLoS ONE. 2013;8(10):e77080 10.1371/journal.pone.0077080 24143203 PMC3797143

[3] Mountain E, Pickles M, Mishra S, Vickerman P, Alary M, Boily M-C. The HIV care cascade and antiretroviral therapy in female sex workers: implications for HIV prevention. Expert Review of Anti-infective Therapy. 2014;12(10):1203–19. 10.1586/14787210.2014.948422 25174997

[4] Evans C, Jana S, Lambert H. What makes a structural intervention? Reducing vulnerability to HIV in community settings, with particular reference to sex work. Global public health. 2009;5(5):449–61. 10.1080/17441690902942472 19507079

[5] Evans C, Lambert H. The limits of behaviour change theory: Condom use and contexts of HIV risk in the Kolkata sex industry. Culture Health & Sexuality. 2008;10(1):27–41. 10.1080/13691050701561393 18038279

[6] Blankenship KM, Friedman SR, Dworkin S, Mantell JE. Structural interventions: concepts, challenges and opportunities for research. J Urban Health. 2006;83(1):59–72. Epub 2006/06/01. PubMed Central PMCID: PMC1473169. 10.1007/s11524-005-9007-4 16736355 PMC1473169

[7] Kerrigan D, Kennedy CE, Morgan-Thomas R, Reza-Paul S, Mwangi P, Win KT, et al A community empowerment approach to the HIV response among sex workers: effectiveness, challenges, and considerations for implementation and scale-up. The Lancet. 2014;385(9963):172–85. 10.1016/S0140-6736(14)60973-9 PMC7394498 25059938

[8] Strathdee SA, Wechsberg WM, Kerrigan DL, Patterson TL. HIV Prevention Among Women in Low- and Middle-Income Countries: Intervening Upon Contexts of Heightened HIV Risk. Annual Review of Public Health. 2013;34(1):301–16. 10.1146/annurev-publhealth-031912-114411 23297666

[9] Shannon K, Strathdee SA, Goldenberg SM, Duff P, Mwangi P, Rusakova M, et al Global epidemiology of HIV among female sex workers: influence of structural determinants. The Lancet. 2014;385(9962):55–71. 10.1016/S0140-6736(14)60931-4 PMC4297548 25059947

[10] Johnston D, Deane K, Rizzo M. The political economy of HIV. Review of African Political Economy. 2015;42(145):335–41.

[11] Jana S, Basu I, Rotheram-Borus MJ, Newman PA. The Sonagachi Project: a sustainable community intervention program. AIDS education and prevention: official publication of the International Society for AIDS Education. 2004;16:405–14. 15491952 10.1521/aeap.16.5.405.48734

[12] Basu I, Jana S, Rotheram-Borus MJ, Swendeman D, Lee SJ, Newman P, et al HIV prevention among sex workers in India. J Acquir Immune Defic Syndr. 2004;36(3):845–52. Epub 2004/06/24. PubMed Central PMCID: PMC2826108. 15213569 10.1097/00126334-200407010-00012 PMC2826108

[13] Poundstone KE, Strathdee SA, Celentano DD. The Social Epidemiology of Human Immunodeficiency Virus/Acquired Immunodeficiency Syndrome. Epidemiol Rev. 2004;26:22–35. 10.1093/epirev/mxh005 15234945

[14] Rhodes T. The 'Risk Environment': a framework for understanding and reducing drug-related harm. Int J Drug Policy. 2002;13:85–94.

[15] Kerrigan D, Moreno L, Rosario S, Gomez B, Jerez H, Barrington C, et al Environmental–Structural Interventions to Reduce HIV/STI Risk Among Female Sex Workers in the Dominican Republic. Am J Public Health. 2006;96:120–5. 10.2105/AJPH.2004.042200 16317215 PMC1470438

[16] Overs C, Loff B. Toward a legal framework that promotes and protects sex workers' health and human rights. Health and Human Rights. 2013;15(1):E186–96. 25006086

[17] O'Laughlin B. Trapped in the prison of the proximate: structural HIV/AIDS prevention in southern Africa. Review of African Political Economy. 2015;42(145):342–61.

[18] Wasserheit JN, Aral SO. The Dynamic Topology of Sexually Transmitted Disease Epidemics: Implications for Prevention Strategies. The Journal of Infectious Diseases. 1996;174(Supplement 2):S201–S13. 8843250 10.1093/infdis/174.supplement_2.s201

[19] Halperin DT, Mugurungi O, Hallett TB, Muchini B, Campbell B, Magure T, et al A Surprising Prevention Success: Why Did the HIV Epidemic Decline in Zimbabwe? PLOS Med. 2011;8(e1000414). 10.1371/journal.pmed.1000414 21346807 PMC3035617

[20] WTO. Trade Policy Review: Report by the Government of Zimbabwe. 14 September 2011. Available online: https://www.wto.org/english/tratop_e/tpr_e/tp352_e.htm. Accessed: 06 Feb 2016: 2011.

[21] IMF. Public Information Notice: IMF Executive Board Concludes 2009 Article IV Consultation with Zimbabwe. May 6, 2009 [cited 2017 Jan]. Available from: https://www.imf.org/en/News/Articles/2015/09/28/04/53/pn0953.

[22] Hanke SH. Zimbabwe: From Hyperinflation to Growth. The Cato Institute: Development Policy Analysis [Internet]. 2008 [cited 2017 Jan]; 6. Available from: https://www.cato.org/publications/development-policy-analysis/zimbabwe-hyperinflation-growth.

[23] Zimbabwe Overview. [Internet]. World Bank. [cited 20 December 2013]. Available from: http://www.worldbank.org/en/country/zimbabwe/overview

[24] Zimbabwe National HIV and AIDS Estimates 2013. Harare: Zimbabwe Ministry Of Health And Child Welfare. Retrieved from http://www.nac.org.zw/documents/documents-and-reports. Accessed: Jun 2016.

[25] Muchini B, Benedikt C, Gregson S, Gomo E, Mate R, Mugurungi O, et al Local Perceptions of the Forms, Timing and Causes of Behavior Change in Response to the AIDS Epidemic in Zimbabwe. AIDS and Behavior 2011;15(2):487–98. 10.1007/s10461-010-9783-z 20803064 PMC3514977

[26] Elmes J, Nhongo K, Ward H, Hallett T, Nyamukapa C, White PJ, et al The Price of Sex: Condom Use and the Determinants of the Price of Sex Among Female Sex Workers in Eastern Zimbabwe. Journal of Infectious Diseases. 2014;210(suppl 2):S569–S78. 25381377 10.1093/infdis/jiu493 PMC4231645

[27] Standing H. AIDS: conceptual and methodological issues in researching sexual behaviour in Sub-Saharan Africa. Soc Sci Med. 1992;34(5):475–83. 1604353 10.1016/0277-9536(92)90202-2

[28] Jewkes R, Morrell R, Sikweyiya Y, Dunkle K, Penn-Kekana L. Transactional relationships and sex with a woman in prostitution: prevalence and patterns in a representative sample of South African men. Bmc Public Health. 2012;12:325 Epub 2012/05/04. PubMed Central PMCID: PMC3433345. 10.1186/1471-2458-12-325 22551102 PMC3433345

[29] Hunter M. The changing political economy of sex in South Africa: The significance of unemployment and inequalities to the scale of the AIDS pandemic. Social Science & Medicine. 2007;64(3):689–700. 17097204 10.1016/j.socscimed.2006.09.015

[30] Silbereisen RK, Tomasik MJ. Mapping Demands of Social Change. 2011. Available from: http://www.llakes.org/wp-content/uploads/2011/02/21.-Silbereisen-Tomasik-final-reduced.pdf.

[31] Gregson S, Garnett GP, Nyamukapa CA, Hallett TB, Lewis JJC, Mason PR, et al HIV Decline Associated with Behavior Change in Eastern Zimbabwe. Science. 2006;311:664–6. 10.1126/science.1121054 16456081

[32] Hawken MP, Melis RDJ, Ngombo DT, Mandaliya K, Ng'ang'a LW, Price J, et al Part time female sex workers in a suburban community in Kenya: a vulnerable hidden population. Sex Transm Infect. 2002;78:271–3. 10.1136/sti.78.4.271 12181465 PMC1744494

[33] Kapiga SH, Sam NE, Shao JF, Renjifo B, Masenga EJ, Kiwelu IE, et al HIV-1 epidemic among female bar and hotel workers in northern Tanzania: risk factors and opportunities for prevention. JAIDS. 2002;29:409–17. 11917247 10.1097/00126334-200204010-00013

[34] Nagot N, Ouangre A, Ouedraogo A, Cartoux M, Huygens P, Defer MC, et al Spectrum of commercial sex activity in Burkina Faso: Classification model and risk of exposure to HIV. JAIDS. 2002;29:517–21. 11981369 10.1097/00126334-200204150-00013

[35] Kitzinger J. Introducing Focus Groups. BMJ: British Medical Journal. 1995;311(7000):299–302. 7633241 10.1136/bmj.311.7000.299 PMC2550365

[36] Bourdillon MFC. The Shona peoples: an ethnography of the contemporary Shona, with special reference to their religion. Rev. ed. Gweru Zimbabwe: Mambo Press; 1982. 344 p.

[37] Strauss AL, Corbin JM. Basics of qualitative research: techniques and procedures for developing grounded theory 2nd ed Thousand Oaks, Calif.: Sage Publications; 1998.

[38] Campbell C, Nhamo M, Scott K, Madanhire C, Nyamukapa C, Skovdal M, et al The role of community conversations in facilitating local HIV competence: case study from rural Zimbabwe. Bmc Public Health. 2013;13:354 Epub 2013/04/18. PubMed Central PMCID: PMC3637528. 10.1186/1471-2458-13-354 23590640 PMC3637528

[39] Attride-Stirling J. Thematic networks: an analytic tool for qualitative research. Qualitative Research. 2001;1:385–405.

[40] Braun V, Clarke V. Using thematic analysis in psychology. Qualitative Research in Psychology. 2006;3(2):77–101.

[41] Oliver DG, Serovich JM, Mason TL. Constraints and Opportunities with Interview Transcription: Towards Reflection in Qualitative Research. Social forces. 2005;84(1273–1289):1273–89. 10.1353/sof.2006.0023 PMC1400594 16534533

[42] Muleya D. Zimbabwe, British firm locked in diamonds war. Zimbabwe Independent. 7th January 2005. Available from: http://www.newzimbabwe.com/pages/mines12.14845.html. [cited 23 December 2013].

[43] Farira D. Eerie silence at Zimbabwe mine. BBC News [Internet]. 4 December 2008. Available from: http://news.bbc.co.uk/1/hi/world/africa/7761268.stm. [cited 20 December 2013].

[44] Fritz K, Woelk G, Bassett M, McFarland W, Routh J, Tobaiwa O, et al The Association Between Alcohol Use, Sexual Risk Behavior, and HIV Infection Among Men Attending Beerhalls in Harare, Zimbabwe. Aids and Behavior. 2002;6(3):221–8.

[45] Kalichman S, Simbayi L, Kaufman M, Cain D, Jooste S. Alcohol Use and Sexual Risks for HIV/AIDS in Sub-Saharan Africa: Systematic Review of Empirical Findings. Prevention Science. 2007;8(2):141–51. 10.1007/s11121-006-0061-2 17265194

[46] Wilson D, Chiroro P, Lavelle S, Mutero C. Sex worker, client sex behaviour and condom use in Harare, Zimbabwe. AIDS Care. 1989;1:269–80. 10.1080/09540128908253032 2488289

[47] Wilson D, Sibanda B, Mboyi L, Msimanga S, Dube G. A pilot study for an HIV prevention programme among commercial sex workers in Bulawayo, Zimbabwe. Soc Sci Med. 1990;31:609–18. 2218643 10.1016/0277-9536(90)90097-c

[48] Schur N, Mylne A, Mushati P, Takaruza A, Ward H, Nyamukapa C, et al The effects of household wealth on HIV prevalence in Manicaland, Zimbabwe–a prospective household census and population-based open cohort study. Journal of the International AIDS Society. 2015;18(1). 10.7448/IAS.18.1.20063 PMC4655223 26593453

[49] Jewkes Rachel, Dunkle Kristin, Nduna M Shai NJ. Transactional Sex and HIV Incidence in a Cohort of Young Women in the Stepping Stones Trial. Journal of AIDS & Clinical Research. 2012;3(158).

[50] Wojcicki JM. Commercial sex work or ukuphanda? Sex-for-money exchange in Soweto and Hammanskraal area, South Africa. Culture Medicine and Psychiatry. 2002;26(3):339–70. 10.1023/a:1021291922026 12555904

[51] Mojola S. Situating transactional sex: Comparisons, history and theory. STRIVE Consortium Learning labs [Webinars] [Internet]. 2014. Available from: http://strive.lshtm.ac.uk/resources/situating-transactional-sex-comparisons-history-and-theory-sanyu-mojola.

[52] Heise L. What works to prevent partner violence: An evidence overview. Working Paper. STRIVE Research Consortium, London School of Hygiene and Tropical Medicine, London. 2011. Downloaded from: http://researchonline.lshtm.ac.uk/21062. Accessed 25 June 2016.

[53] O'Brien S, Broom A. Gender, culture and changing attitudes: experiences of HIV in Zimbabwe. Culture, Health & Sexuality. 2013;15(5):583–97. 10.1080/13691058.2013.776111 23520999

[54] Moyo ON, Kawewe SM. The Dynamics of a Racialized, Gendered, Ethnicized, and Economically Stratified Society: Understanding the Socio-Economic Status of Women in Zimbabwe. Feminist Economics. 2002;8(2):163–81.

[55] Robinson J, Yeh E, World Bank. Transactional sex as a response to risk in Western Kenya. American Economic Journal: Applied Economics. 2011;3:35–64.

[56] Desmond N, Allen CF, Clift S, Justine B, Mzugu J, Plummer ML, et al A typology of groups at risk of HIV/STI in a gold mining town in north-western Tanzania. Soc Sci Med. 2005;60(8):1739–49. 10.1016/j.socscimed.2004.08.027 15686806

[57] van den Borne F. "I am not a prostitute": Discords in targeted HIV/AIDS prevention interventions in urban and trading centers in Malawi. Series on HIV/AIDS in Sub-Saharan Africa: Sex, Gender and Policy 2003;Working Paper Series, 13: 8. Available: https://cdn1.sph.harvard.edu/wp-content/uploads/sites/114/2012/10/RP214.pdf. Accessed: 06 Feb 2016.

[58] Ngugi EN, Wilson D, Sebstad J, Plummer FA, Moses S. Focused peer-mediated educational programs among female sex workers to reduce sexually transmitted disease and human immunodeficiency virus transmission in Kenya and Zimbabwe. J Infect Dis. 1996;174:S240–S7. 8843254 10.1093/infdis/174.supplement_2.s240

[59] Gregson S, Adamson S, Papaya S, Mundondo J, Nyamukapa CA, Mason PR, et al Impact and process evaluation of integrated community and clinic-based HIV-1 control: a cluster-randomised trial in eastern Zimbabwe. PLOS Medicine. 2007;4:e102 10.1371/journal.pmed.0040102 17388666 PMC1831737

[60] Singh K, Sambisa W, Munyati S, Chandiwana B, Chingono A, Monash R, et al Targeting HIV Interventions for Adolescent Girls and Young Women in Southern Africa: Use of the PLACE Methodology in Hwange District, Zimbabwe. AIDS Behav. 2009;14:200–8. 10.1007/s10461-009-9572-8 19452272 PMC3966072

[61] Maher L, Mooney-Somers J, Phlong P, Couture M-C, Stein E, Evans J, et al Selling sex in unsafe spaces: sex work risk environments in Phnom Penh, Cambodia. Harm reduction journal. 2011;8(1):1–11. 10.1186/1477-7517-8-30 PMC3339327 22099449

[62] Mishra S, Steen R, Gerbase A, Lo Y-R, Boily M-C. Impact of high-risk sex and focused interventions in heterosexual HIV epidemics: a systematic review of mathematical models. PLOS ONE. 2012;7:e50691 10.1371/journal.pone.0050691 23226357 PMC3511305

[63] Gregson S, Gonese E, Hallett TB, Taruberekera N, Hargrove JW, Lopman B, et al HIV decline in Zimbabwe due to reductions in risky sex? Evidence from a comprehensive epidemiological review. Int J Epidemiol. 2010. 10.1093/ije/dyq055 PMC2972436 20406793

[64] Lugalla J, Emmelin M, Mutembei A, Sima M, Kwesigabo G, Killewo J, et al Social, cultural and sexual behavioral determinants of observed decline in HIV infection trends: lessons from the Kagera Region, Tanzania. Social Science & Medicine. 2004;59(1):185–98. 15087153 10.1016/j.socscimed.2003.10.033

[65] Cheater AP, Gaidzanwa RB. Citizenship in Neo-Patrilineal States: Gender and Mobility in Southern Africa. Journal of Southern African Studies. 1996;22(2):189–200.

