# Supplementary material for: A reconfiguration of the sex trade: How social and structural changes in eastern Zimbabwe left women involved in sex work and transactional sex more vulnerable
Source: PLoS One. 2017 Feb 22;12(2):e0171916. doi: 10.1371/journal.pone.0171916 (PMC5321466; doi:10.1371/journal.pone.0171916)
Supplement: S1 Text — All quotes that are used in the main text are supplied in full and without edits (the texts remain anonymized). (DOC) [file pone.0171916.s001.doc]

**Supplementary material, S1**

Below are the unedited quotes as they appeared in the transcripts; spelling, grammar and punctuation have not been corrected (identifying information remains omitted). These are listed in the order in which they appear in the main manuscript. If a quote in the main text was formed by removing some intervening text or quotes, the intervening text has been included here.

Male, RTS

Daniel: “I think I am now a bit more mature and I have quite some experience, I started working when things were still okay, I started working late 80s and early 90s, I would say that we had our piece of cake, we really enjoyed ourselves, but nowadays if you ask all the men here when they last stop a sex worker or a girlfriend, it is long back, very long back. People use to do it long back when the economy was still okay. Sex work is going down, it was just resuscitated for a short time during the Chiadzwa diamond days, but generally the way we are now living is that sex work has down”

FSW, growth point

Hannah: “But the time of the Diamond Panners, we really made money, because they would pay to about US$100- 150 per session.”

KN: “ZAR100 or US$100, what are you saying?”

Hannah: “US100! The diamonds panners had a lot of money; they didn’t even bother or care about money. They would even give you such an amount for free, without even bothering, or even throw it at you without asking for sex.”

FSW, growth point

Esther and Charity: “Aah these days it’s difficult, people no longer have money, it’s by lucky”

Esther: You are lucky to get someone who will pay you ZAR50,

KN: Only ZAR50?

Esther: Yes, you can’t refuse because the state you will be in at home, you will have to accept that amount, sometimes even as little as enough to buy a bundle of vegetables.

Male, small town

Hugh: “nowadays there is no longer month end as far as things are concerned, it depends on what you earn on that day, it can be an income that you managed to get on that day, it can be grocery, be it a bar of soap, cooking oil, mealy-meal, gadget or whatever you have on that specific day, these girls accept anything you have as payment.”

Male, RTS

Gilbert: “You may not even have planned this but suppose you get into a bar and there are many women in the bar, say in [names of places known for sex work], I do not have any intentions at all, but because I get drunk then I begin dancing with them, my eyes and my thinking will send me into it, I end up having sex with them”

Male, SFA

Chris: “They would come to drink, there were women who would go to the beer hall just to drink, for those who came just for drinking, they will buy their beer and drink it outside the bar, usually on the veranda. But there were those women who would buy their beer and drink it in the bar till late, from 6pm till even 7pm or so, then you would know that they are not just for drinking beer but they are up to something else. For someone to be involved in this sex work business, it means you have a lot of money, so people will invite each other and go for a beer binge drinking. Today he has money then he will call a lot of friends to go and drink beer, that’s what these young men do, but nowadays money is scarce, for someone to get money and go and drink it, it’s rare because life is now very tough, he would rather stay at home.”

Male, estate

Brian: “We have one more bar here but at the moment it’s not functional but when it is functioning a lot will be happening here. It is in the same room with the TV, the scenario is that people will be coming here to drink and to watch TV, watching TV does not select whether it’s male or female, people just come to watch TV. A lot of arrangements and agreements will take place then, people that’s where they meet each other, talk and arrange for sex in the TV room.”

KN: “So is this bar currently functioning or do people just come to watch TV?”

Brian: “The bar is not functioning at the moment, people just come to watch TV.”

KN: “The bar is not functioning?”

Brian: “The bar is not functioning due to financial constraints”

Males, RTS

KN: “What about women who used to go into the bars, were there any women who would go into the bars in this area?”

Walter: “Yes there used to be women here who were entering the bars, we ended up chasing away women from the bars, we didn’t want women anymore in the bars”

KN: “Was it a unanimously agreed thing that as a community you agreed not to entertain women in the bars or what?”

Walter: “To answer your question. I would say that many people died. People had a nasty experience due to this disease, so they do not want it to befall them. At funerals people will be aware of the at person’s previous’ activities and if s/he was not straight, then they warn those remaining to be watchful for their time will be very close, so people are afraid and they exercise self-control.”

Peter: “Plus these women they used to document all the partners they had slept with, so they will leave a record that is listing all people who used to sleep with her”

KN: “Who would know of what was in the record?”

Michael: “The whole village, the list will be read in public”

KN: “What if your wife was there, she would hear about that”

Michael: “of course, she will know of all your hidden activities”

KN: “But I think it helped a lot”

Walter: “Yes it helped; it made men change their attitudes”

FSWs, small town

Grace: “there are also those who are selling their items from the flea markets or are vendors, yet they are also sex workers”

Barbara: yes they will be selling their commodities, they pretend to be going to the toilet to relieve themselves, yet they will be going to have sex for a short time. These are sex workers are even doing this business more than us who will wait for sunset to go to the bars; these women would have been in business the whole day and in the evening again, so they are doing it more than us who are well known.”

Grace: “and for all the clients they get them, for those who have failed to get transport, these women are the first to know them and get them, for those who are stranded and are confused to know what they want, they are again the first ones to know and get them. So they make more money than us the registered ones. I say we are registered because we are well known, we are even known by the police that we are commercial sex workers. But for those who are doing sex work and do not even get into the bars and the like, they are the most dangerous and they are the ones who are fuelling the spread of HIV/AIDS. Pure prostitutes who are doing it on full time, they use condoms, no matter what. Even if they know that they are now HIVAIDS positive, they know the rules and they use condoms strictly. But for those who are doing it outside, they are tricky, they do not use condoms, people will be imagining that they are still health again because the partnership will be sometimes on the bases of whether they will get married, they are not so sure where the relationship is going to end, so they do not use condoms, as long as they are single. Sometimes for someone who will be in Harare may fall in love with a girl here whom he won’t know her true character, such people are the ones who spread HIV/AIDS on a fast rate, because they are still trusted. As for us we are no longer trusted and we now have a number plate, no one trusts us anymore.”

FSW, small town

Grace: “for us it’s no longer profitable for us to go to the bars, we now has to find means of making money, if a sex work gets money, she will leave sex work, there is no more money from the bars as it used to be long back. So most sex workers now rely on self-help jobs to make money, the money they use to make from the bars is no longer readily available, everyone is facing financial hardships”

Male, estate

KN: “How was it [sex work] done in the past?”

Paul: “In the past, women use to fill the bars and men will fight over women in the bars, again at work places where people have contracts they would frequent there. Nowadays they are no longer doing that, you find them doing sex work from their homes and from the work place, not in the bars as before. If you go to the bars you would think that there are no sex workers here yet there are there, but they are doing it in their homes and in the bushes, not in the bars anymore.”

KN: “okay, so it’s no longer as popular in the bars as it used to be?”

Paul: “Yes they no longer go to the bars, but long back women use to fill the bars and again young women use to line up outside the bars at the verandas and at the windows of the bars”

KN: “why would they line up outside the bars, they will be afraid?”

Paul: “Yes they will be afraid of entering the bars. Men will just come out of the bar and have their pick from these lined up girls. Like in the cities where by girls will be wearing nothing except dust coats and will be flashing for men to pick them, it’s no longer common, they are doing it from their homes”

FSW, estate

KN: “Where do men go to find women for sex in here in Selbourne?

Marian: “They meet in the bars here [TV room] usually”

KN: “After work?”

Marian: “Yes usually after work, as people within the same locality, arrangements on where to meet are made and as soon as people knock off from work they go to the agreed place and have sex there.”

KN: “How do people make those arrangements, are there signals that go around indicating that they are in agreement?”

All laughing

Mary: “I will give an example of the haulage truck drivers that come to load timber here. They find a go between to send and find a woman for them to sleep with. This guy who is sent we will be working together, he tells me then I will go and sleep with that driver in his truck.”

KN: “Do you slip out from work to go and sleep with him during working hours?”

Mary: “no as soon as I break from work say around 11pm, I go there and we have sex in the truck till the morning.”

Male, SFA

KN: “So how do you identify sex workers from a group besides what you have mentioned? Like you mentioned the way they talk the way they dress, for sure one would have dressed to attract men, besides these, are there other ways, yes Max?”

Max: “There are situations that I will be aware of the behavior of such a person so no matter how she dress I know that she is approachable because she is a sex worker, because I will be aware of her behavior”

KN: “How do you know it, from friends or what?

Max: “Yes people share and they may nickname her Bacoss or thirst-quencher”

FSW, SFA

Christina: “Yes, nowadays, it is rare that we meet clients at the beer halls. We meet them at our homes, people no longer go to the beer halls.”

Sylvia: “We also see each other at the traditional ceremonies to mark the death of a departed family member (chenura)”

Christina: “And the consolation ceremonies (nyaradzo)”

Sylvia: “It is usually done over night, the activities that are done during the night.”

KN: “How do you do it?”

Sylvia: “We just act…”

KN: “Suppose you attended a chenura, how will you do it to get the attention of a partner, do they just approach you or you make a certain action for them to see you?”

Sylvia: “I dance,”

KN: “okay,…..”

Sylvia: “From the way I dance, they will definitely notice me”

Married woman, RTS

Margaret: “Men no longer have money and people are also afraid of dying. HIV is killing and people are now afraid of the infection. So they refrain from seeking sex worker’s services. The sex workers also are no longer doing it as frequent as possible; they get into the business when there is need, like once a month”

Vendors, RTS

KN: okay, but is there a specific category of men that you can say are popular in doing se work, say teachers or something, that maybe popular in engaging in sex work?

Gloria: “It was common with men who were involved in illegal mining of diamonds, the Chiadzwa time; yes that’s when it was common that any man who will be coming from there will be looking for sex workers.”

KN: “so they were solely from Chiadzwa?”

Nancy: “Yes, those were the days that sex work was very popular, nowadays if you sleep with a man, what you get, nothing, so it is no longer profitable.”

Mavis: “again, nowadays, the sex workers are concentrated at that organization there that is distributing grain”.

KN: “okay there is an organization in this area that they target?”

Mavis: “Yes, the sex workers are targeting truck drivers that are bringing in trucks of food there, so they target them”

Victoria: “even these shop assistants, they know very well the dynamics that are going on there. They are the ones that are sent to call sex workers by these truck drivers. They tell you. If you go there they will definitely tell you because they are the ones who play with the sex workers.”

Males, RTS

KN: “okay, is there a specific time or season that sex workers can say that they are very busy and that they are maybe making a lot of money, yes tell us Walter?

Walter: “in this area there are a lot of activities going on in this area, when there are activities that generate money, for instance weekends, there are a lot of activities going on, then that’s the chance they have to make money, especially at the knock off time of people’s activities”

GC: “so can we say weekends?”

Walter: “yes weekends and sometimes during pay days and at shut down of companies, the number of sex workers increase here”

Jeffrey: “the other thing that happens is that some of these sex workers know people’s pay days, they know the companies and the pay days, so they usually grab the opportunity”

KN: are there a lot of companies in this area?

Jeffrey: “not really but we have some non-governmental organizations operating in here, so they keep a record of that”

Males, RTS

GC: “What about nowadays, how much are they [sex workers] charging?”

Peter: “Nowadays the market is low, payments are low, ever since the mine was closed, these girls are getting very little…”

Someone: “If they get groceries then they are paid. They get a piece of bath soap. They can be given a bottle of cooking oil or anything as a form of payment”

FSWs, estate

KN: “What are the times that you likely or most women are likely to work? The times you say that business is at its best!”

Phoebe: “like here at [name of forestry estate], we get our diamond at the end of the month, that’s when we pay each other”

KN: “What diamond are you talking about, salary?”

Phoebe: “a hamper that we are given as part and parcel of the salary.”

KN: “okay you get a hamper on monthly bases!”

Phoebe: “Yes. If they take two months without paying us, then we will be having sex with each other for those two months for nothing. So that’s the deal”

KN: “so when the man gets his hamper he then pays you before the wife receives the hamper?”

Chloe: “he has to pay me before taking the hamper groceries home, once it reaches home to his wife, I won’t get anything.”

GC: “So you would have agreed to sleep with that person, whether he is time pusher or a permanent or a casual client, you agree that at the end of the month when he gets his diamond, he should pay you the first before the hamper gets home. ….

Phoebe: “2 litres…”

KN: “And that you agree that we you sleep together then get the payment when you all get paid, before he takes the hamper home to his wife…..”

Phoebe: “that’s correct…”

GC: “as soon as he gets the hamper he has to pay you whether he is married of not, be it 2litres or a bar of soap, he has to give you first before he takes everything home”

Phoebe: “yes usually 2litres of cooking oil and a bar of soap…”

Chloe: “or 4litres and a bar of soap”

GC: “so does not this create squabbles and problems for instance the man realize that after taking what is due to you he remains with very little to take to his family, maybe you would have sex the previous week and it will now be paying for something that has passed long back, we all know that it is not easy for someone to pay a debt, what if he decides to slip away without paying you, to go and give his family everything, doesn’t this create a lot of fights between you and him that everybody will end up knowing what is happening?”

Phoebe: “if he runs away without paying then know definitely that he will not come back again for such services, he will have to look for someone else not me”

KN: “so you do not make a follow up at all?”

Phoebe: “no I will not make a follow up, it just means the end of the relationship”

KN: “there won’t be any fights again?”

Phoebe: “If I am tricked, I am tricked; it is not possible to make a follow up, is it?”

Chloe: “I will try to make a follow up aah!”

KN: “yes Chloe, tell us!”

GC: or maybe you just want to make a follow-up thinking that maybe he had forgotten

Claris: “what happens is that, you may say that you do not want to make a follow up; he would have used me for nothing so I will make a follow up until he pays me my money or what we have agreed. As soon as he pays me, I won’t bother him anymore or even to bother his new partner, I won’t, I will leave him and look for someone else.”

KN: “so what happens here is that people have sex for the whole month on the basis of credit, then when they get paid at the end of the month. How do you keep the record of the payments that are due to you, do you write them down? How do you do it?”

Phoebe: “what happens is that we get paid by these partners on the bases of what we get here as payment on the end of the month. Suppose we are given only 4litres of cooking oil plus 5bars of soap, and then you can’t expect a lot. But I may see you being given 20litres of cooking oil, so do you think I will accept as little as 2litres, I will ask for more. So the payments depend on how much we are paid here as our salaries.”

FSW, growth point

Esther: “What happens with these older women are also very jealous of the younger girls; they will tell the men in the bar that e younger women are infected by HIV/AIDS, so men shouldn’t pick them”

FSW, estate

Chloe: I would say that both people can approach each other but it’s usually the woman, take for instance I meet a guy at a church or funeral gathering or any gathering, when I see a guy that I fancy, the moment I look at him, there is a touch, when I greet him, there is something that must go on. When I go to greet him, a decently married woman would just politely greet someone and may say, I am actually coming from a funeral and it will be true, but as for me I will greet him seductively, then that guy will know that I am free to approach. I also use to be bothered before I was a sex worker on how then do someone know that I am approachable. You know what there those very large earrings, ring were like but they are large, they are called “do not hesitate to approach me!” When you are wearing those earrings, men will know that you are approachable. The way I will talk to a man for instance at a bus terminus, he should know that I am free, I can go and approach him and ask him seductively like say, was there any bus that has passed so far, (standing up to demonstrate, twisting her body), the way I will be standing my posture also reflects that I am asking for more. If I am a decent person, I will be talking to him more politely, ‘my son-in law was there any transport here, I am coming from a funeral’, politely. It’s a different way altogether when I am a sex worker, the guy I will be talking to will definitely know that I am free.

(All laughing from the gestures)

Chloe: it is called marketing yourself

Married TS, growth point

KN: “These women who are doing TS, do they consider themselves as sex workers or what?”

Tiffany: “They will be thinking that they are fending for their families”

KN: “But do they consider this as sex work?”

Tiffany: “when we meet as women and we are rebuking each other about this behavior, one will tell you that, the man you are chiding me about brings food at home, my own husband doesn’t bring food for the children, that’s why chose to do this because all is going well for me”

KN: “okay so do they consider this as sex work like the one done by the ladies who do sex work in the bars?”

Tiffany: “They do not see it as sex work at all”

KN: they do not consider themselves as sex workers?

Tiffany: “Yes, even if my husband is told at the beer hall that I was seen doing sex work, then he comes home and ask me, I will refuse everything and tell him I do not know anything”

KN: “okay, so what form of payment do they get from the boyfriends?”

Tiffany: “What we see from those who are doing it, it is perfect for them everything moves on well for them, the husband does nothing he is just seated at home doing nothing, so the wife will go like last time there was Chiadzwa, for those who were going to Chiadzwa, she will go for two days, three days, she comes back with lots of groceries or blankets, then you ask people coming there, have you seen so and so, they tell you no, we did not see her, she would have gone with the diamond panniers and after a few days she brings a lot of goods, so you see that everything moves on well for them even the family looks well”

KN: so are they given money, groceries or other goods?

Tiffany: “like last the past time, the time of the diamond panniers, they were given money”

KN: “cash?”

Tiffany: “cash, us as next doors we will be seeing that for sure there is cash”

Males, small town

KN: “so what exactly makes a man to approach the woman, are there any signs or any non-verbal cues or any communication that maybe going on that shows that the woman is inviting you? What really would be happening, I do not know, but is it easy just to approach any woman whom you meet along the road?”

(All laughing)

Charles: “usually it is in the way these woman talk to you, it will be in such a way that they are interested in you”

KN: “so that makes a man then to approach the woman? Yes Moses”

Moses: I will refer back to the two categories of sex workers; there are those whom I can call money driven and right now we have poverty, poverty driven. For those who are poverty driven they maybe a married couple and the man cannot support their families, the wife then can approach you in a manner of asking for grain/maize or anything to feed their families in favor, they will have sex, for sex, they will agree to have sex with you. Then there are those who go into the bars or pubs and are willing to have sex in return for money. These are the categories that we usually meet. But it is due to poverty that we find most women being driven into prostitution.”

KN: “okay, what happens over the issue of payment, say in an instance where the woman has been forced by poverty to do sex work, the grain that you pay her is sufficient for the sex services that she provides or you add some extra money?”

Moses: “usually, it depends with the men how much he considers the poverty of the woman. Sometimes the man might just decides to help her for free of charge, there are some who say well, we have sex”

Males, SFA

Chris: “still on these young people, the young girls. Parents try their best to reprove them and instruct them not to hang around with elderly men but they do the opposite. If I go to the shopping centre, these young girls are not even hesitant to ask money or things from us, you will hear them saying, “T[name]’s father, now things are expensive, may you buy me a soft drink, maputi etc”, without even respect, they have a way of talking that you end up proposing her because she is too used to you. You may tell others that this girl is getting too close to me, and then they will tell you that she loves you, so we end up proposing and end up sleeping together. He didn’t have the intention at all, but because the child is insisting”

FSW, estate

KN: “okay, typically how young do women/ girls start sex work?”

Phoebe: “these days at 12 years, they are starting sex work”

KN: “12 years?”

Phoebe: “yes! 12 year olds are now an eye sore in the bars”

KN: “do these children have parents?”

Phoebe: “Parents have nothing to say about it, they are failing to control them”

GC: “What about the elders or authorities, police or guards, who come to the bars, don’t they chastise these children? Usually the elders will chase out these children”

Phoebe: “Nowadays, you won’t find any authority in the bars, there are no police officers anymore manning the bars nowadays. When I was still a girl, I had my sister who uses to go to the bars. She will leave her baby with me, I would follow her to the bar and when I got to the door of the bar, there use to be guards there manning the door, at N[name] bar. I couldn’t get into the bar to get my sister. The guard asked me what I wanted from the bar; I told her I wanted to see my sister. But nowadays there are no more police authorities controlling anyone at the bars”

KN: “how about elders in the bars?”

Phoebe: “to say to them go back home? Ah, impossible!”

KN: Beatrice

Beatrice: “it’s impossible now, these children are totally impossible nowadays, these children now depend from what they get from men from the bars. That’s where they get sugar, soap, and all other basics.”

KN: “so do the parents receive and accepts these groceries even if she knew that they are from men from the bars?”

Beatrice: “yes she will accept them”

Males, estate

Brian: “Some people still have a wrong perception about condoms. There are still some people who believe that condoms are for commercial sex workers. So as a result if someone falls for somebody’s wife they think that she doesn’t have any infection, they do not use a condom and they also believe that a condom is only used to a sex worker. Again a married woman would not prefer a condom to be used for her for she doesn’t consider what she is doing as sex work.”

KN: “okay she doesn’t consider herself as a sex worker…..?”

Brian: “yes, she just considers herself as a married person as soon as you produce a condom, she will ask: ‘are you taking me as a prostitute that you want to use a condom with’…..”

KN: “so it’s the women who refuse to use the condom”

Brian: “yes…….”

Vendors, small town

KN: “okay, one more question, for the married women who are sex workers, do you think it is common knowledge to the police just like it is with those sex workers who are doing it publicly?”

Judith: “They do not know about it. But these types of women are the major carriers of HIV/AIDS. They spread the disease on a faster rate than normal sex workers. Men will be going out with them thinking that they are the only partners besides this woman’s husband, yet there will be a lot of men believing the same. So the disease spreads rapidly, for if someone goes to a known sex worker, there they use protection because they will know that she is a commercial sex worker but with these married women they may not even use protection.”

KN: “so what is your view over sex work and sex workers? I think everyone should give her views for this is the last question as we come to the end of the program”

Violet: “I am pained by married women who are doing sex work than with those who are doing full time sex work in the bars. These married women are spreading HIV/AIDS like a veldt fire and it’s a concern to us, but for those who are in the bars doing commercial sex work, those kinds of people use protection, I do not know what can be done about them for these are the trouble causers.”

Married women, estate

KN: “Okay. If you are to look at the two categories of women, on the issue of risks, What do you think are the main differences between open commercial sex workers and women who sell sex discretely especially when looking at the risk of getting infected by HIV/AIDS or other diseases?”

Rosemary: “the married women are more at risk of getting the infection, the open sex workers are free to use condoms and because of the nature of their jobs, they are very free to use condoms, be it short time or what, they are consistent in their condom use. The married women do not have that flexibility, suppose they meet and the opportunity avails itself, then they do not have a condom both of them, they will do it without a condom because they have limited opportunities and they contract the disease easily”

KN: “what do others say?”

Katie: “as has been said by Rosemary, I would say that the married women are more at risk because suppose I have a john who is satisfying me sexually, sometimes john will be using a condom with me but when I go back to my husband, we will not be using a condom, suppose he has been sleeping with Mary, maybe without a condom, it can also be the other way round, I would priorities grocery that I receive from John and I agree to have unprotected sex with him, so I will be getting the disease both from home and from John, the boyfriend. The sex worker who is doing it professionally, she will be aware of the fact that sex work is her job therefore she does it cautiously and always use a condom, it is not the same case with us who are married.”

GC: “But for those sex workers from the bars, do they have a partner whom they really trust or who are kind of on a permanent basis?”

KN: “Situations where your husband may have a girlfriend for quite a long period say from fife months and above? Do they end up using condoms or not”

Katie: “that’s the issue we were talking about, they can go along together and it’s a situation that I will be taking from John, my husband will be bringing the disease from Mary while I am also bringing it from John, sometimes this Mary will also be seeing another James while Mary is also seeing other men, James, my own boyfriend John maybe having his own wife and another girlfriend, Midoo, …….”

All laughing

GC: then it becomes a chain

KN: “which group is more likely to use condoms, between women doing TS and those who go to the bars and do it publicly? Let’s hear from others”

Lucy: “those who are at freedom are sex workers form the bars. They will be cautious to always carry a condom because they will be aware that they will definitely meet someone there who will be willing to sleep with them, so they always move around with a condom knowing that they will meet such a situation.”

KN: okay, yes that’s right, what do others say over the issue, do you agree with what Lucy has just said. Yes Rose?

Rosemary: “I agree with what Lucy has said because if I as a married woman bring in a condom into my bedroom, my husband will fight with me, he will question me where I got such ideas. He will complain that he cannot enjoy a sweet wrapped in the wrappers after he had paid bride price for me so he would not even agree to that so we do it unprotected, so sex workers are free to use condoms at any time they want either male or female condoms, so they are at freedom. So for me to say that I have been to the clinic and i heard that there are female condoms, then he will slap me.”
