# Supplementary material for: A reconfiguration of the sex trade: How social and structural changes in eastern Zimbabwe left women involved in sex work and transactional sex more vulnerable
Source: PLoS One. 2017 Feb 22;12(2):e0171916. doi: 10.1371/journal.pone.0171916 (PMC5321466; doi:10.1371/journal.pone.0171916)
Supplement: S2 Text — (DOCX) [file pone.0171916.s002.docx]

**Supplementary quote, S2**

MN: “What types of clients do you meet, are they locally based or what?”

Charity: “They are mixed, some are local, some will be coming from places such as Harare and other places. They will be trying to get to their destinations, so we catch them”

Catherine: “Others just hear about girls here and the come looking for a specific person”

KN: “Would you have met before?”

Esther : “No, if there is some activity going on like the diamond panning that was there last year, the clients tell each other and they will come with specific names. You will hear them asking for you and you know there and then that cash has arrived for you.”

(FSWs, growth point )
