# Supplementary material for: A reconfiguration of the sex trade: How social and structural changes in eastern Zimbabwe left women involved in sex work and transactional sex more vulnerable
Source: PLoS One. 2017 Feb 22;12(2):e0171916. doi: 10.1371/journal.pone.0171916 (PMC5321466; doi:10.1371/journal.pone.0171916)
Supplement: S3 Text — (DOCX) [file pone.0171916.s003.docx]

**Supplementary quote, S3**

Gilbert: “You may not have planned it, I would not have had any intentions at all, but suppose I go to a bar say in [names of places known for sex work], and there are many women, because I get drunk I begin dancing with them. Then my eyes and my thoughts will drive me to have sex with them.” (Male, RTS)
