# Supplementary material for: A reconfiguration of the sex trade: How social and structural changes in eastern Zimbabwe left women involved in sex work and transactional sex more vulnerable
Source: PLoS One. 2017 Feb 22;12(2):e0171916. doi: 10.1371/journal.pone.0171916 (PMC5321466; doi:10.1371/journal.pone.0171916)
Supplement: S4 Text — (DOCX) [file pone.0171916.s004.docx]

**Supplementary quotes, S4**

*KN: “Is it easy to negotiate condom use, do you face any challenges in negotiating condom use … what’s your view Catherine?*

*Catherine: “We do meet some men who do not want to use condoms. Usually these men would be infected by diseases, but would say, “I will pay you more [for unprotected sex]”. And if we refuse and he notices that you are serious [insist on condom use] then he would say, “I was only joking!” If you are a clever person do not agree to have sex with them for these are the types of men that will pierce a condom.” (FSW, growth point)*

*KN: “Okay now let’s focus on condoms, do men use condoms when they have sex with sex workers?”*

*Noah: “What usually happens with condom use is that men use condoms, but as soon as they get drunk they rarely use condoms, some even remove condoms when they are having sex with these ladies and they do it without protection.” (Male, small town)*
