# Supplementary material for: A reconfiguration of the sex trade: How social and structural changes in eastern Zimbabwe left women involved in sex work and transactional sex more vulnerable
Source: PLoS One. 2017 Feb 22;12(2):e0171916. doi: 10.1371/journal.pone.0171916 (PMC5321466; doi:10.1371/journal.pone.0171916)
Supplement: S5 Text — (DOCX) [file pone.0171916.s005.docx]

**Supplementary quotes, S5**

Grace, in a discussion on the shifting earning potential of sex workers, reports that the lack of demand for sex work has driven some women to seek additional sources of income.

Grace: “For us it’s no longer profitable for us to go to the bars, we now have to find a means of making money. If a sex worker gets money, she will leave sex work, there is no more money from the bars as it used to be long back. So most sex workers now rely on self-help jobs to make money, the money they used to make from the bars is no longer readily available, everyone is facing financial hardships” (FSW, small town)

This was corroborated by males who indicated that FSWs would sell items they had bought with earnings during the boom period.

KN: “How much could would women sell sex for during the Chiadzwa diamonds wave?”

Moses: “About $100-150US for the whole night, the money was plenty”

KN: “So did they make a lot of money?

Moses: “They made money, but now they are beginning to sell those things they bought from that time.”

(Male, small town)
