# Supplementary material for: A reconfiguration of the sex trade: How social and structural changes in eastern Zimbabwe left women involved in sex work and transactional sex more vulnerable
Source: PLoS One. 2017 Feb 22;12(2):e0171916. doi: 10.1371/journal.pone.0171916 (PMC5321466; doi:10.1371/journal.pone.0171916)
Supplement: S6 Text — (DOCX) [file pone.0171916.s006.docx]

**Supplementary quotes, S6**

Mary: “I will give an example of the haulage truck drivers that come to load timber here. They find a go-between [third party] to send and find a woman for them to sleep with. This go-between will be my work colleague, he gives me the message and then I will go and sleep with that driver in his truck … as soon as I break from work, at around 11pm, and we have sex in the truck till the morning.” (FSW, estate)

Paul: “As we are working we definitely talk and we agree on meeting each other later after work. Some of course utilize the break time to do that.”

(Male, estate)
