# Supplementary material for: A reconfiguration of the sex trade: How social and structural changes in eastern Zimbabwe left women involved in sex work and transactional sex more vulnerable
Source: PLoS One. 2017 Feb 22;12(2):e0171916. doi: 10.1371/journal.pone.0171916 (PMC5321466; doi:10.1371/journal.pone.0171916)
Supplement: S7 Text — (DOCX) [file pone.0171916.s007.docx]

**Supplementary quotes, S7**

Susan: “Of course [prostitution has always been there] but it wasn’t [the same] as nowadays where we see a 12 year old child already going to the bars or a ten year child now knows that if she is given money by so-and-so, she can now go and [buy] something. It wasn’t as before, nowadays it’s too much. I do not know whether back then children were dull and maybe nowadays children are too clever, I do not know.” (Vendor, small town)

KN: “If you are to make an assessment of your area, at what age do children, young girls start engaging in sex work?”

Christina: “They start at around 11years”

KN: “Do these children have parents?”

Christina: “Some have parents, some are orphans; as you must be aware, most of the children are now orphans”

KN: “But for those who have parents, what do the parents say about it? Do they no longer control and discipline their children?”

Christina: “Some parents encourage their children to do it because they bring sugar [basics] home. They will be actually fending for the whole family, so parents do not restrain them.”

Sylvia: “Things are quite expensive nowadays.” (FSWs, SFA)
